# A rare case of oral myiasis in a non-tropical region: the role of systemic vulnerability

**DOI:** 10.1590/S1678-9946202668026

**Published:** 2026-03-13

**Authors:** Gökçe Kızılkale Kayıkcı, Ayşe Yılmaz, Furkan Arabacı, Hale Ahsen Yardibi Demir, Mesut Özsoy

**Affiliations:** 1 Kastamonu Training and Research Hospital, Department of Anesthesiology and Intensive Care, Kastamonu, Turkey; 2 İzmir Tınaztepe University, Faculty of Medicine, Department of Anesthesiology and Reanimation, İzmir, Turkey; 3 Kastamonu Training and Research Hospital, Department of Medical Microbiology, Kastamonu, Turkey; 4 Kastamonu Training and Research Hospital, Intensive Care Nursing Unit, Kastamonu, Turkey

**Keywords:** Oral myiasis, Cirrhosis, Malnutrition, Intensive care unit

## Abstract

Myiasis is a rare parasitic infection caused by the larvae of *Diptera* flies, which infest the tissues of humans or animals and are typically found in warm, humid climates. Oral myiasis is uncommon in healthy individuals and typically occurs when adult flies lay eggs or larvae near the mouth or on open wounds. Malnutrition, immunodeficiency, poor oral hygiene, dental problems, neurological or psychiatric conditions, and alcoholism are the main risk factors. In this report, we present a case of oral myiasis that occurred in a patient with multiple comorbidities, including malnutrition, immunological and neurological dysfunction (lung carcinoma, cirrhosis, cachexia, Parkinson's disease). The patient was admitted to our intensive care unit while intubated because of confusion, respiratory failure, and sepsis, in which oral myiasis was identified. Due to the patient's comorbid conditions, ivermectin could not be administered. A conservative approach was used, including daily cleaning with diluted hydrogen peroxide and povidone–iodine, together with mechanical removal of the larvae. The infestation was completely resolved within three days. This case shows that oral myiasis may develop in non-tropical settings when systemic vulnerability exists. It highlights the importance of regular oral exams and mouth care for high-risk patients.

## INTRODUCTION

Myiasis is a rare parasitic infection caused by fly larvae that invade human or animal tissues. The flies responsible belong to the order Diptera, with approximately 80 species known to infest human tissue^1^. Oral myiasis is uncommon in healthy individuals and usually occurs when adult flies lay eggs or deposit larvae near the mouth or on open wounds. Although it is rare in modern healthcare, the condition still occurs most often in tropical and subtropical regions^2^.

Human myiasis usually occurs on intact or damaged skin but can also affect the ears, nose, eyes, paranasal sinuses, lymph nodes, anus, vagina, and oral cavity^2^. Oral forms are among the rarest types of the disease^3^. It usually affects patients who are immunosuppressed, unconscious, intubated, malnourished, or have poor oral hygiene. Trauma, malignancy, advanced age, and chronic systemic diseases are among the main predisposing factors^1-3^.

Our case is notable because it happened in a non-tropical region where myiasis is rare. The patient had several risk factors, including immunodeficiency, malnutrition, neurological dysfunction, necrotic oral tissue, and long-term intubation, all of which probably created a suitable environment for larval infestation.

Even with modern medical care, unexpected infestations may occur when host defenses are weakened, underscoring the need to recognize such rare events. Informed consent for publication, including the use of clinical data and photographs, was obtained from the patient's relatives.

### Ethics

Written informed consent for publication (including clinical images) was obtained from the patient's relatives.

## CASE REPORT

An 82-year-old man with chronic obstructive pulmonary disease (COPD), cirrhosis, Parkinson's disease, and lung carcinoma was found unconscious at home and brought to the emergency department (ED) by his relatives. On arrival, his Glasgow Coma Scale (GCS) score was 7/15, and he showed severe respiratory distress. Chest computed tomography (CT) showed upper-lobe emphysema typical of COPD and right lower-lobe consolidation with air bronchograms, suggesting pneumonia. He was diagnosed with pneumosepsis and remained intubated in the ED for three days before being transferred to the intensive care unit (ICU).

**Figure 1:**
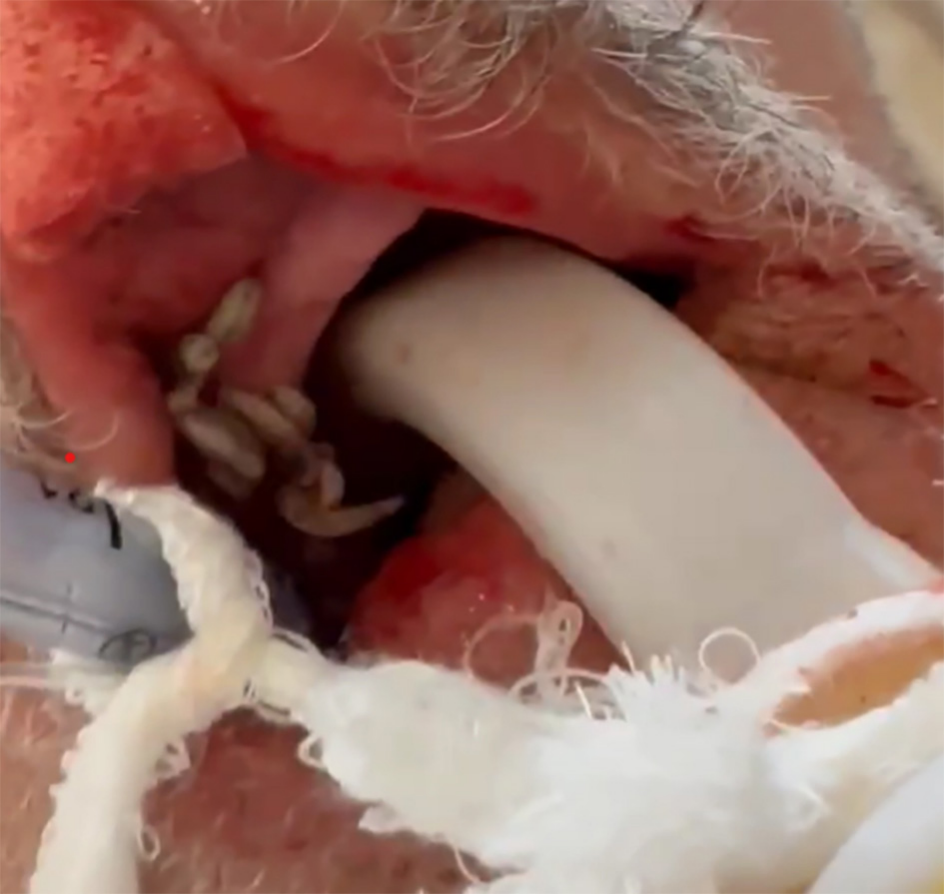
Clinical presentation of oral myiasis.

On ICU admission (hospital day 4), his vital signs were stable: temperature 36.4 °C, blood pressure 130/80 mmHg, heart rate 92/min, and SpO_2_ 94% on volume-controlled ventilation. During the initial examination following stabilization, multiple motile larvae were observed in the patient's oral cavity. Closer inspection revealed their location in the maxillary vestibule, surrounded by erythematous and necrotic tissue (Figure 1). The patient appeared cachectic, with a body mass index (BMI) of 16.2 kg/m^2^. Blood tests revealed pancytopenia with marked lymphopenia (white blood cell (WBC) count 1.72 × 10^9^/L, lymphocyte count 0.14 × 10^9^/L, hemoglobin 10.0 g/dL, platelet count 38 × 10^9^/L), together with clear signs of malnutrition (albumin 2.3 g/dL, prealbumin <3 mg/dL). Inflammatory markers were high, with CRP at 109 mg/L and procalcitonin (PCT) at 2.56 ng/mL. We sent blood, urine, and tracheal aspirate to the microbiology laboratory. Cultures remained negative.

**Figure 2:**
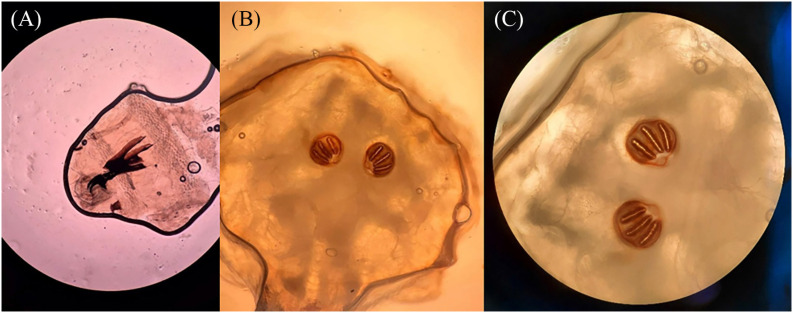
Light microscopic images of larval structures: (A) Cephalopharyngeal skeleton, examined at ×40 magnification; (B) Posterior spiracles, examined at ×40 magnification; (C) Posterior spiracles showing three straight slits and a complete peritreme, examined at ×100 magnification, morphologically consistent with second and third-instar larvae of the Calliphoridae family.

In total, two larvae measuring approximately 9 × 2 mm were submitted for parasitological examination. One larva was kept at room temperature to observe development to the adult stage, while the other was used for microscopic examination. For microscopy, the larva was briefly immersed in boiling water, fixed in 80% ethanol, and cleared in 5% potassium hydroxide (KOH) for 24 hours. The specimen was examined under light microscopy, focusing on the cephalopharyngeal skeleton and posterior spiracles, which showed three straight slits and a complete peritreme, consistent with second and third-instar larvae of the Calliphoridae family (Figure 2).

**Figure 3:**
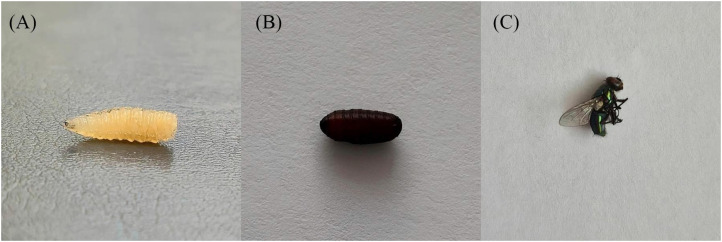
Life cycle progression of the maggots retrieved from the patient: (A) Third-instar larva at the time of removal; (B) pupa formed after incubation in the laboratory; (C) adult fly, consistent with a Calliphoridae species (*Lucilia* spp.), emerging on day 7.

Further molecular identification was not performed; however, based on morphological findings and clinical context, the larvae were morphologically consistent with *Lucilia* spp. Under laboratory conditions, the remaining larvae pupated within three days and reached the adult stage by day seven (Figure 3).

In the ICU, we performed bedside mechanical debridement and daily irrigation with diluted hydrogen peroxide (1–1.5% from 3%) and povidone-iodine to minimize mucosal toxicity while ensuring eradication. Paranasal sinus CT and endoscopic examination ruled out any deeper invasion. Ivermectin was not given because the patient had advanced cirrhosis. Empiric meropenem was started on admission. Within three days, PCT decreased from 2.56 to 0.81 ng/mL, and the WBC count increased from 1.72 to 4.11 × 10^9^/L. Severe thrombocytopenia was treated with platelet transfusions, after which counts stabilized. Hypoalbuminemia was managed with intravenous albumin. Supportive care included cirrhosis-adapted fluid therapy, prophylaxis, and enteral nutrition.

Although oral myiasis was completely eradicated within three days and did not recur, his overall clinical condition remained poor due to the multiple comorbidities. Despite continued intensive care, the patient died on ICU day 39.

## DISCUSSION

Oral myiasis is a rare parasitic infestation in modern intensive care settings^2^. Although it is traditionally considered a disease of tropical regions, recent European evidence indicates that hospital-acquired infestations can also occur in temperate, high-income countries, suggesting that systemic vulnerability rather than environmental exposure may be the key determinant. These cases have been described even in intensive care units with strict infection control and oral hygiene protocols, suggesting that prolonged mechanical ventilation, neurological dysfunction, and impaired host defense play a decisive role in the pathogenesis of this condition^4,5^.

Our patient had cirrhosis-related immune dysfunction (CAID), sepsis-induced immunosuppression, advanced age, denture use, and malnutrition. We believe the combination of these factors created a favorable environment for larval infestation. In addition, motor impairment due to Parkinson's disease may have negatively affected oral hygiene, while dysphagia and sialorrhea may have increased secretion stasis^6^. Mucosal trauma related to denture use may also have provided a suitable site for larval implantation^7^.

Cirrhosis and sepsis impair host defenses through distinct yet synergistic mechanisms. Cirrhosis primarily compromises innate immunity by weakening neutrophil function, reducing complement activity, and impairing opsonization^8^. In contrast, sepsis (especially in its advanced stages) induces a secondary immunodeficiency state characterized by lymphopenia and myeloid dysfunction^9^. The pancytopenia observed in our patient reflects this profound immune suppression. Moreover, protein-energy malnutrition, evidenced by low albumin and prealbumin levels and cachexia, likely increased the susceptibility to infection and delayed mucosal healing, creating a permissive environment for larval infestation^10^.

In line with this view, a recent systematic review on myiasis associated with malignant wounds reported that myiasis often occurs in patients with malignancy, chronic tissue damage, and poor nutritional status, and is frequently underestimated in clinical practice^11^. Although the review focused on cutaneous myiasis, the underlying risk factors are similar to those observed in our patient. This supports the view that oral myiasis should not be considered an isolated infestation, but rather a marker of systemic vulnerability and impaired host defense.

Only a few cases of oral myiasis have been reported from Turkey^12-15^, and these were mainly managed with mechanical debridement and local irrigation, with systemic ivermectin rarely used. In other countries, ivermectin is more commonly included in treatment plans^1,2^. In our patient, we avoided systemic ivermectin due to advanced cirrhosis and the potential hepatotoxicity of the drug. Instead, daily mechanical debridement combined with diluted hydrogen peroxide (H_2_O_2_) and povidone-iodine irrigation was applied. To prevent further irritation of the necrotic, erythematous oral mucosa and to reduce mucosal toxicity, we preferred approximately 1–1.5% H_2_O_2_ instead of the conventional 3% solution^16^. Additionally, the broad-spectrum antibiotic therapy initiated for pneumosepsis may have prevented secondary bacterial infection arising from necrotic oral tissues, a measure generally recommended in reported cases of myiasis to avoid secondary infection and local spread^2,7^.

The effects of oral myiasis are not limited to the oral cavity. Larval activity may cause local ulceration, necrosis, and secondary infection. In some cases, invasion has been reported to extend to the paranasal sinuses, orbit, or even the intracranial area^1,15,17^. Migration of larvae into the oropharynx may result in aspiration or airway obstruction. When deeper invasion is suspected, paranasal sinus CT and endoscopic evaluation are recommended^1,2^. Timely exclusion of sinus or orbital involvement is important to prevent life-threatening complications. Considering this possibility, we confirmed by imaging that the lesion in our patient was confined to the oral cavity.

The larvae were detected on the fourth day of hospitalization, after the patient had been intubated and treated in the emergency department for respiratory failure. Based on the rapid larval development and the patient's clinical course, the infestation most likely began before ICU admission or during the early emergency period. This suggests that the case falls between community- and hospital-acquired categories and highlights the importance of early oral examination and preventive mouth care in critically ill patients. Oral myiasis is rare but is likely underreported in non-tropical regions. Moreover, climate change and the northward spread of tropical fly species may increase the number of cases in temperate areas in the future. Therefore, systematic case reporting is recommended^4^.

## CONCLUSION

Oral myiasis should be considered not only a local infestation but also a warning sign of systemic frailty and impaired host defense. In high-risk patients, maintaining awareness and providing simple, regular oral care can significantly reduce the risk of this condition.

## Data Availability

The authors will not make additional data available due to ethical restrictions related to patient confidentiality.
